# Awake Bruxism Treated With Quetiapine in a Patient With Alzheimer’s Dementia

**DOI:** 10.1192/bjo.2025.10738

**Published:** 2025-06-20

**Authors:** Regina Rachel Khakha, Seema Rani

**Affiliations:** 1 ESIC Medical College & Hospital, Faridabad, India; 2 University College of Medical Sciences, Delhi, India

## Abstract

**Aims:** Bruxism is a stereotyped movement disorder of tooth grinding or clenching. Unlike sleep bruxism, awake bruxism is not a sleep disorder, but is secondary to disorders of the central nervous system, such as Parkinson’s disease, stroke and advanced dementia. We report a case of debilitating awake bruxism that developed during the course of Alzheimer’s dementia, unrelated to neuroleptic use.

**Methods:** A 63-year-old man presented to our outpatient clinic of a tertiary care hospital in India, with a 4-year history of progressive short-term memory loss, increasing apathy and a constant audible teeth grinding in the day, which distracted him from social interaction. He was not taking any medications. Examination demonstrated phasic teeth grinding and extensive teeth wearing, but no extrapyramidal features or transient hypertonicity induced by distraction. Mini-Mental State Examination (MMSE) score was 15 out of 30. The psychometric testing was consistent with moderate dementia of Alzheimer’s type. Computed tomography revealed age-related cerebral atrophy.

**Results:** Donepezil 5 mg was initiated and subsequently increased to 10 mg for Alzheimer’s dementia improved MMSE to 20 three months after commencement. Quetiapine was prescribed for the bruxism. The patient reported a complete disappearance of awake bruxism at a daily dose of quetiapine 100 mg, with no occlusal appliances.

The biochemical origin of bruxism involves complicated interactions of various neurotransmitters. A central role of the dopaminergic system in awake bruxism was suggested from clinical observations in patients with Parkinson’s disease and attenuation of symptoms with dopaminergic medications. A favourable response to quetiapine in the present patient suggests that bruxism in dementia might also involve the dopaminergic pathway.

**Conclusion:** A 63-year-old male treated with 100 mg daily doses of quetiapine for Alzheimer’s dementia experienced a significant reduction of awake bruxism. More studies are needed to determine whether quetiapine has a long-term effect against awake bruxism.

